# *Plesiomonas shigelloides* Bacteremia: A Scoping Review of Epidemiology, Clinical Characteristics, Outcomes, and Implications of Antimicrobial Stewardship

**DOI:** 10.3390/pathogens15010123

**Published:** 2026-01-22

**Authors:** Nur Izzatul Auni Romli, Salina Mohamed Sukur, Kumutha Malar Vellasamy, Kartini Abdul Jabar

**Affiliations:** 1 Department of Medical Microbiology, Faculty of Medicine, Universiti Malaya, Lembah Pantai, Kuala Lumpur 50603, Malaysia; nurbromli@gmail.com (N.I.A.R.); kumuthamalar@um.edu.my (K.M.V.); 2 Kementerian Kesihatan Malaysia, National Institutes of Health (NIH), No. 1, Jalan Setia Murni U13/52, Seksyen U13, Bandar Setia Alam, Shah Alam 40170, Malaysia; drsalina_msukur@moh.gov.my

**Keywords:** *Plesiomonas shigelloides*, bacteremia, bloodstream infection, mortality, epidemiology, predisposing factors

## Abstract

*Plesiomonas shigelloides*, an aquatic Gram-negative bacillus often associated with self-limiting gastroenteritis, has been reported worldwide. However, to date, no reviews have specifically investigated *P. shigelloides* bacteremia, which is rare and potentially fatal. This scoping review aimed to examine the existing literature to identify the epidemiology, clinical characteristics, antimicrobial susceptibility, and outcomes of *P. shigelloides* bacteremia. A PRISMA-ScR-guided search of PubMed, Scopus, Web of Science, and Embase identified 22 published cases, all reported as single-patient case reports. Cases were globally distributed, with the majority reported from the Americas and Europe. The median patient age was 46 years. The case fatality rate was 27.3% (*n* = 6/22). Most patients had identifiable host risk factors, particularly hematological disorders, neonatal status, or immunocompromised status, and environmental exposure such as raw seafood consumption or contact with freshwater. Clinical presentations were heterogeneous, commonly including fever and sepsis or septic shock. Microbiologically, *P. shigelloides* demonstrated consistent intrinsic resistance to ampicillin while retaining susceptibility to multiple antimicrobial classes. Poor outcomes were more closely associated with host factors and delayed presentation than with antimicrobial resistance. Early diagnosis, targeted therapy, and antimicrobial stewardship are essential for optimizing outcomes in this rare but severe infection.

## 1. Introduction

*Plesiomonas shigelloides* (*P. shigelloides*) is an oxidase-positive, facultatively anaerobic Gram-negative bacillus that belongs to the order *Enterobacterales* and represents the only species within the genus *Plesiomonas*. This organism is widely distributed in aquatic environments, including freshwater, estuaries, and soil, and is frequently isolated from fish, shellfish, crustaceans, amphibians, and reptiles [1]. In humans, *P. shigelloides* is best known as an enteric pathogen, associated with self-limiting gastroenteritis following ingestion of contaminated water or raw or undercooked seafood [1,2]. Although most infections manifest as mild diarrheal illness, a growing body of literature demonstrates that *P. shigelloides* can cause invasive and life-threatening extraintestinal disease.

Bacteremia due to *P. shigelloides* is rare, but when it occurs, it is frequently associated with severe clinical outcomes, including septic shock, multiorgan failure, and death [3,4]. The first case report was conducted in 1973 [5]; however, mortality was determined to be caused by other complications linked to sickle cell disease. By contrast, evidence has shown that, with delayed treatment, *P. shigelloides* bacteremia can be fatal compared to uncomplicated gastrointestinal infection, especially in high-risk populations [1,4], including neonates, people with hematological disorders, or other immunocompromised states. 

Host-related risk factors appear consistently across published cases and appear central to disease pathogenesis. Iron overload states, including primary hemochromatosis, thalassemia, and sickle cell disease, are disproportionately represented among patients with invasive *P. shigelloides* infection [6,7]. Experimental and clinical evidence indicate that *P. shigelloides* possesses iron-acquisition systems, including heme utilization pathways, that enhance virulence in iron-rich environments [8].

General reviews were conducted in the 1990s regarding *P. shigelloides* infection [9,10], but no comprehensive narrative synthesis focused specifically on *P. shigelloides* bacteremia.

Existing reviews often aggregate enteric and extraintestinal infections or are limited by dated literature, incomplete case capture, or a lack of systematic methodology. Given the continued emergence of atypical presentations, potential emerging antimicrobial resistance, and diagnostic methods, a more recent and structured overview of *P. shigelloides* bacteremia is warranted.

A scoping review is particularly well-suited to this topic as it allows for the systematic mapping of heterogeneous evidence derived primarily from case reports and small observational studies. 

This scoping review aimed to collate peer-reviewed research published in the literature to clarify the epidemiology, host risk factors, clinical manifestations, antimicrobial susceptibility patterns, and outcomes of *P. shigelloides* bacteremia. This study identifies knowledge gaps and patterns that are not apparent in earlier case reports, providing direction for clinical awareness. By synthesizing evidence across decades of research, this review aimed to enhance understanding of this rare but important infection.

## 2. Methods

### 2.1. Search Strategy and Data Sources

The protocol for this scoping review was developed in accordance with The Preferred Reporting Items for Systematic Reviews and Meta-Analyses Extension for Scoping Reviews (PRISMA-ScR). The protocol was registered on Open Science Framework (Registration DOI: 10.17605/OSF.IO/M64X9).

Literature searches were conducted across four databases: PubMed, Web of Science, Embase, and Scopus. The keyword “*Plesiomonas*” was combined with outcome-related terms using AND, while related terms (“bacteremia” OR “bloodstream infection”) were combined using OR, with parentheses applied to maintain a consistent query structure across databases. Full search strategies are provided in Supplementary Materials Table S2. The searches were conducted on 8 December 2025, and the articles were exported into EndNote 21 for deduplication of search results and screening support. A completed PRISMA-ScR checklist, as well as the key terms used, are provided in Supplementary Materials Tables S1 and S2.

### 2.2. Study Selection

Inclusion criteria for this review are as follows: (1) *Plesiomonas* bloodstream infection; (2) human patient; (3) case report, case series, or cohort studies; (4) full-text available; and (5) written in English. No year filter was used for this review. All retrieved records were imported into EndNote, and duplicates were removed prior to screening. Titles and abstracts were screened independently by two authors to identify potentially relevant studies, followed by a full-text review to confirm eligibility. Screening was performed manually rather than using dedicated platforms such as Covidence or Rayyan, due to the small number of included studies. Data extraction was independently performed by the authors using a standardized Excel spreadsheet, with discrepancies resolved by consensus to ensure consistency and minimize bias. Extracted data included epidemiology, host risk factors, clinical manifestations, antimicrobial susceptibility patterns, and outcomes of *P. shigelloides* bacteremia. A narrative synthesis was conducted for this scoping review.

## 3. Results

### 3.1. Characteristics of Included Studies

**Figure 1:**
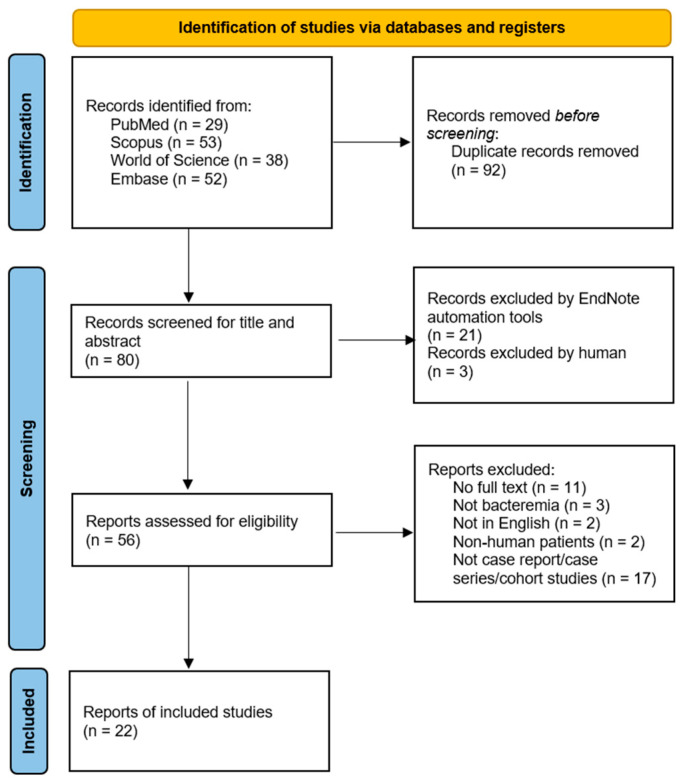
PRISMA flow chart for case reports included.

A total of 29, 53, 38, and 52 records were identified from PubMed, Scopus, Web of Science, and Embase, respectively. After excluding 92 duplicate records, 80 unique articles remained. Titles and abstracts were initially screened using a combination of automated and manual approaches. Automated screening was performed in EndNote 21 by creating a smart group to identify records containing the keywords “*Plesiomonas*” in the title or abstract, as well as the common misspelling “*Pleisiomonas*” in the title. Records identified through this automated process were subsequently subjected to independent manual screening of titles and abstracts by two reviewers to confirm relevance. This resulted in the exclusion of 21 articles by EndNote and 3 articles by the reviewers. A full-text assessment was conducted for the remaining 56 articles, of which 21 met the inclusion criteria and were included in the final review. One study was included from the references of other studies. These 22 studies were subsequently selected for data extraction and synthesis. The study selection process is illustrated in Figure 1.

A total of 22 cases of *Plesiomonas* bloodstream infection were reviewed and analyzed in this scoping review, all of which were reported as single-patient case reports. No case series or cohort studies were identified.

### 3.2. Study Distribution by Geographic Region

The geographic range of the included studies was analyzed using World Health Organization regions. Of the 22 reported cases of *Plesiomonas* bloodstream infection, the majority were reported from the Americas (54.5%, *n* = 12), followed by Europe (22.7%, *n* = 5). The Western Pacific region accounted for five cases (22.7%, *n* = 5). No cases were identified from the Southeast Asian, Eastern Mediterranean, or African regions.

At the country level, the United States reported the highest number of cases (40.9%, *n* = 9), followed by Canada and Japan (9.1%, *n* = 2 each). Single cases were reported from Australia, Belgium, Brazil, Finland, Germany, Greece, Hong Kong, Malaysia, and Sweden. In total, 22 individual cases were included in the review, and a summary of the cases is provided in Table 1.

### 3.3. Patient Characteristics

A total of 22 published cases of *Plesiomonas* bacteremia were included in the analysis (Table 2). The median age of affected patients was 46 years (interquartile range [IQR]: 15.5–68), spanning pediatric and adult populations, with a male predominance (13/22, 59%) [11,13,14,15,17,18,22,23,24,25,29,30,31]. Overall, 16 patients (72.7%) survived, while 6 patients (27.3%) died [13,14,20,23,24,25].

Analysis of clinical characteristics showed that most patients had identifiable predisposing factors. The most frequently reported form of exposure was consumption of raw or undercooked seafood, documented in eight cases [11,14,15,18,19,24,29,31], followed by freshwater exposure in six cases [13,17,22,24,25,28], highlighting environmental risk factors.

Underlying medical conditions were commonly present. Hypertension [27,28,30,31] and active chemotherapy or cytotoxic therapy [10,16,17,30] were reported in four cases each. Conditions reported in three cases each included splenectomy or functional asplenia [13,20,24], cardiovascular disease [11,25,27], hematological malignancies [10,16,17], chronic liver disease or cirrhosis [22,29,31], iron overload [15,20,24], and pregnancy-associated infections [14,18,26].

Less frequently reported risk factors included hemoglobinopathies, such as thalassemia [20,21] and sickle cell disease [19,21] (where two cases of each were identified), and diabetes mellitus [22,23] (two cases identified). Overall, *Plesiomonas* bacteremia primarily affects individuals who have experienced environmental exposure or have underlying host vulnerabilities, particularly immunocompromised states, liver disease, iron overload, or hematological disorders, with notable mortality highlighting its clinical significance.

### 3.4. Clinical Presentations

Across reported cases of *Plesiomonas* bacteremia, several consistent clinical features were observed. Fever was the most frequently reported symptom, occurring in the majority of cases [10,11,12,13,14,15,16,18,19,20,21,24,30,31], and was often accompanied by other systemic inflammatory signs such as chills [10,11,15,20,24]. Together, these findings indicate that *Plesiomonas* bacteremia presents as a nonspecific febrile illness.

Sepsis and septic shock were observed in 36.3% of reported cases, representing the second-most common clinical manifestation among the published case reports [13,19,20,21,24,25,26,29]. This severe presentation was commonly accompanied by multiorgan failure [19,21,23,24,25], although some patients presented with hypotension without shock [16,28], indicating variability in hemodynamic involvement.

Gastrointestinal manifestations were another recurring feature. Diarrhea [11,12,16,20,21,27], enterocolitis [29], and vomiting [10,11,12,13,19,20,21] were frequently described, consistent with gastrointestinal involvement in many cases. However, gastrointestinal symptoms were absent in a subset of patients.

Hematological abnormalities were commonly reported, though they varied between cases. Anemia [16,17,19,20,21,23,24] and thrombocytopenia [14,16,17,19,20,24] were frequently documented, particularly in patients with severe infections. Disseminated intravascular coagulation (DIC) was reported in a minority of cases [19,20,24]; however, other patients showed no hematological involvement.

Less common organ-specific manifestations were also documented. Neurological involvement, including seizures, encephalitis, or altered mental status, was reported in a smaller number of cases [14,18,31]. Similarly, soft tissue infections were infrequently observed [15,17,28], indicating these presentations were uncommon.

Overall, reported cases demonstrate that *Plesiomonas* bacteremia most commonly presents with fever, systemic inflammatory features, and frequent gastrointestinal symptoms, with substantial variability in severity and organ involvement across cases.

### 3.5. Antimicrobial Susceptibility Patterns

Across reported cases of *Plesiomonas* bacteremia, patterns of antimicrobial susceptibility varied. Consistently high resistance to ampicillin was reported across multiple studies [10,11,13,14,15,16,21,26,29,30]. Resistance to other penicillin-based agents, including ticarcillin [11] and piperacillin [13,30], and third- or fourth-generation cephalosporins [22], was uncommon. Intermediate susceptibility was rarely reported, with limited data indicating intermediate responses to cefazolin [26], amikacin [13], and gentamicin [26] in isolated cases.

In contrast, isolates demonstrated broad susceptibility to fluoroquinolones, which were the most consistently active class, particularly ciprofloxacin [10,16,17,21,22,24,26,27,28,30,31], followed by levofloxacin [24,27,29,30], pefloxacin [20], and ofloxacin [14]. This consistent susceptibility suggests that fluoroquinolones are a reliable treatment option for *Plesiomonas* bacteremia.

Cephalosporins overall showed favorable activity, particularly for second- to fourth-generation agents. Susceptibility was most frequently reported for ceftriaxone [11,21,24,26,27,28,29,30,31], ceftazidime [10,11,16,20,26,29,30], and cefepime [24,27,29,30]. Alternatively, first-generation cephalosporins exhibited more variable susceptibility [11,14,30], indicating they may be less reliable compared to later-generation agents.

Aminoglycosides, especially gentamicin, were commonly active and often used in combination therapy [10,11,14,16,17,21,24]. Other antimicrobial classes generally showed good susceptibility profiles, including β-lactam/β-lactamase inhibitor combinations [16,24,26,27,29,30,31], carbapenems [11,14,17,20,21,24,26,27,28,29,30], monobactams [20,29,30], trimethoprim–sulfamethoxazole [13,14,16,17,21,24,26], tetracyclines [10,13,20], and chloramphenicol [11,17,20].

Overall, this indicates that *Plesiomonas* is intrinsically resistant to ampicillin and shows variable susceptibility to early-generation β-lactams, while remaining broadly susceptible to fluoroquinolones, later-generation cephalosporins, aminoglycosides, and carbapenems. These patterns suggest that fluoroquinolones are an appropriate empiric or targeted therapy, particularly in severe bloodstream infections.

## 4. Discussion

This scoping review synthesizes peer-reviewed case reports on *P. shigelloides* bacteremia, a rare but clinically important invasive organism typically associated with self-limiting enteric disease. The collective findings from the published literature suggest that *P. shigelloides* bacteremia is an illness with variable clinical presentation, host-related predispositions, and a notable burden of morbidity and mortality relative to its incidence. Although limited in number, the reported cases reveal recurring patterns relevant to clinical practice.

The clinical characterization of *P. shigelloides* bacteremia has a central theme across the literature. The vast majority of reported cases occurred in patients with identifiable predisposing conditions, including iron overload (such as hereditary hemochromatosis [15,20,24], thalassemia [20,21], and sickle cell disease [19,21]), chronic liver disease [22,29,31], hematological malignancy [10,16,17], functional or anatomical asplenia [13,20,24], and neonates [14,18,26]. These findings strongly support the classification of *P. shigelloides* as an opportunistic pathogen with an invasive potential that is unmasked in the context of impaired immune defense or altered host physiology.

Iron overload emerges as one of the most striking and consistent risk factors. Multiple case reports document bacteremia in patients with hemochromatosis, often with severe or fatal outcomes. Experimental studies show that *P. shigelloides* possesses iron-acquisition mechanisms, including heme utilization systems, which may enhance their growth and virulence in iron-rich environments [8,32]. This parallels the siderophilic behavior of other aquatic Gram-negative organisms such as *Vibrio vulnificus* and *Yersinia enterocolitica* [33]. The overrepresentation of iron overload states among bacteremia cases suggests that dysregulated iron metabolism contributes to both susceptibility and disease severity [33,34].

Chronic liver disease and cirrhosis represent other major predisposing factors. Bacterial translocation from the gut into the bloodstream is facilitated by cirrhosis-associated immune dysfunction, increased intestinal permeability, and impaired hepatic clearance, particularly following exposure through diet or freshwater [35]. *P. shigelloides* may exploit compromised mucosal barriers and impaired hepatic clearance to establish systemic infection [29]. The frequent coexistence of iron overload in cirrhotic patients may further amplify this risk [36].

Neonatal *P. shigelloides* bacteremia, although rare, is uniformly severe and often complicated by central nervous system involvement. Poor outcomes are observed despite appropriate antimicrobial therapy [14,18,26]. Neonatal immune immaturity [37] and limited physiological reserve could determine this outcome rather than antimicrobial failure. Uncertainties exist regarding acquisition routes in perinatal settings, e.g., transplacental transmission [38], ascending genital tract infection [26], or peripartum exposure [1], and warrant heightened vigilance.

Notably, gastrointestinal symptoms reported in this review were absent or mild in many bacteremia cases (vomiting 31.8% and diarrhea 27.3%), diverging from the organism’s classical enteric presentation. This variability suggests that subclinical intestinal colonization [39] or alternative portals of entry, i.e., the skin or soft tissue [17,28], may precede invasive disease in susceptible hosts, contributing to diagnostic delay and complexity. Polymicrobial infections have also been found in cases of intra-abdominal pathology or traumatic exposure [13,21,23,27,30], suggesting that *P. shigelloides* may occur alongside other aquatic or enteric pathogens. This variability adds to the diagnostic complexity and warrants heightened clinical suspicion for at-risk patients.

It needs to be emphasized that social and environmental determinants of health play a significant role. Homelessness [40], incarceration, lack of access to clean water, and occupational exposure to aquatic environments may intersect with biological risk factors to facilitate infection [41]. These revelations broaden the relevance of *P. shigelloides* bacteremia beyond traditional medical risk groups and emphasize the need for a holistic approach to prevention and management that considers environmental and social contexts.

The findings of this review may inform clinical practice by highlighting the potential diagnostic challenges associated with *P. shigelloides* and its variable presentation. While freshwater and seafood exposure are recognized risk factors [1], bacteremia without preceding gastrointestinal symptoms may be more common than previously appreciated, particularly in patients with host vulnerabilities such as chronic liver disease, malignancy, or iron overload. In these cases, infection may present as occult sepsis, with the absence of gastrointestinal manifestations delaying recognition despite relevant exposure history [15]. Greater awareness of this presentation may prompt broader diagnostic evaluation in at-risk septic patients.

From a microbiological standpoint, *P. shigelloides* demonstrates relatively consistent antimicrobial susceptibility patterns. Intrinsic resistance to ampicillin is well-documented and attributable to beta-lactamase production [42], rendering ampicillin an unreliable therapeutic option. The frequent inclusion of ampicillin in primary susceptibility panels likely reflects testing performed before species-level identification rather than an expectation of clinical efficacy [43]. Most isolates included in this review were susceptible to third-generation cephalosporins [10,11,14,16,20,21,24,26,27,28,29,30,31], fluoroquinolones [10,16,17,21,22,24,26,27,28,30,31], carbapenems [11,14,17,20,21,24,26,27,28,29,30], aminoglycosides [10,11,14,16,17,21,24], and beta-lactam/beta-lactamase inhibitor combinations [16,24,26,27,29,30,31]. However, available evidence is derived from a limited and heterogeneous body of case reports spanning multiple decades and regions and may not fully capture geographic or temporal shifts in resistance or exclude emerging patterns [44].

The overall fatality rate for *P. shigelloides* bacteremia in the published case reports was 27.3% (6/22), where five of the six deaths (83.3%) occurred in the male patient subgroup (5/13, 38.5%), suggesting higher mortality among men within the cohort. Fatal outcomes were most frequently observed among neonates [14], patients with iron overload [20,24], or post-splenectomy status [13,20,24], likely due to delayed diagnosis, rapid progression, and limited physiological reserve. Alternatively, favorable outcomes were more common in immunocompetent hosts [12] or when early recognition and targeted therapy were achieved [19,22,28]. This reinforces the importance of prompt microbiological identification and supportive care. However, these mortality estimates are derived from a small number of heterogeneously published case reports and lack denominator data, limiting their epidemiological interpretation. Case reports are also more likely to document severe or fatal presentations, potentially inflating the observed mortality. Consequently, they likely overestimate true mortality and cannot be generalized to the broader population; instead, they represent the severe end of the clinical spectrum.

Comparative analysis indicates that mortality is primarily driven by host vulnerability and clinical severity at presentation rather than antimicrobial resistance. Asplenia presented most frequently in 50% of fatal cases but was absent among survivors, while iron overload (33.3% vs. 6.3%) and freshwater exposure (50% vs. 18.8%) were also more common in non-survivors. Markers of severe illness further distinguished these groups, with higher rates of septic shock (66.7% vs. 25%), multiorgan failure (50% vs. 12.5%), and disseminated intravascular coagulation (33.3% vs. 6.3%) observed among fatal cases. Together, these findings suggest that, in high-risk hosts, *P. shigelloides* bacteremia may progress rapidly and result in poor outcomes despite in vitro antimicrobial susceptibility. Although based on limited data, these findings highlight the value of early clinical detection in vulnerable populations.

Moreover, reported cases from temperate climates and non-endemic regions [11,12,13,14,15,17,19,22,23,24,25,26,27,28,29] challenge the perception of *P. shigelloides* as a pathogen restricted to tropical or subtropical settings. The apparent predominance of cases from the Americas and Europe likely reflects disparities in diagnostic capacity, surveillance systems, and publication practices rather than true geographic risk [45]. Underrepresentation from regions such as Southeast Asia, the Eastern Mediterranean, and Africa may be attributable to underdiagnosis [45], limited microbiological testing [45], and lower publication rates [46]. These patterns have important clinical implications, as a lack of regional familiarity with the pathogen may contribute to delayed recognition in cases of Gram-negative sepsis.

Despite increased awareness from this review, important knowledge gaps persist. The incidence of *P. shigelloides* bacteremia is likely underestimated due to underreporting, misidentification, and limited routine testing in many laboratories [1]. While advances such as MALDI-TOF and molecular diagnostics could improve recognition [47], inconsistent microbiological reporting limits robust assessment of management strategies and prognostic factors. In addition, data on adjunctive therapies, source control, and preventive measures in high-risk populations are lacking, and the absence of comparative studies precludes evidence-based recommendations. Systematic data collection, collaborative case reporting, and inclusion of *P. shigelloides* in Gram-negative bacteremia surveillance networks are needed to address these gaps [1]. Furthermore, as a scoping review of published case reports, this study is methodologically limited by an inability to establish causality or provide definitive population-level incidence and outcome data.

In summary, *P. shigelloides* bacteremia is a rare but severe infection that disproportionately affects vulnerable populations. A valid interpretation of the available data is limited by small case numbers, regional underrepresentation, and incomplete microbiological reporting, reinforcing the need for cautious interpretation and improved surveillance.

## 5. Conclusions

*P. shigelloides* bacteremia remains a rare but clinically relevant cause of severe infection. Evidence from published case reports suggests that this bacteremia has intrinsic resistance to ampicillin and preserved susceptibility to multiple other antimicrobial classes, supporting targeted therapy once the organism is identified. However, the evidence is derived from a small and heterogeneous body of case reports, often involving severe presentations, which limits our ability to form firm conclusions regarding prognosis or outcome determinants. Disease severity appears more closely related to host vulnerability and acuity at presentation than to antimicrobial failure. These findings reinforce the importance of early recognition, antimicrobial stewardship, and cautious interpretation of outcomes based on rare, case-based evidence.

## Figures and Tables

**Table 1:** Summary of all case reports included in this study.

Study	Country	Age	Gender	Relevant Medical Background	Complications	AMR Patterns	Survival Outcomes
[11]	USA	59	Male	Osteoarthritis: Treated with fenoprofen Supraventricular tachycardia: Treated with propranolol Right total hip replacement Exposure: Ingested oysters, fish, scallops, shrimp	Nausea Vomiting Diarrhea Tenesmus Fever Chills Leukocytosis Painful, swollen left ankle Arthrocentesis: Elevated leukocytes	Susceptible: CFZ, FOX, CXM, CAZ, CTX, CHL, IPM, GEN, TOB Resistant: AMP, TIC	Recovered
[12]	Finland	15	Female	Visited Leningrad, Russia	Fever (39 °C) Vomiting Multiple watery diarrhea episodes Frontal headache Dehydration	Susceptible: TMP-SDZ	Recovered
[13]	USA	32	Male	On hemodialysis for 8 years—chronic glomerulonephritis Two failed renal transplants Severe dialysis access difficulties Exposure: Recent rafting (Delaware River) Allergy: all antibiotic classes except chloramphenicol	Fever (39.4 °C) Nausea Vomiting Collapse Epigastric pain Right upper quadrant pain Shock Leukocytosis Metabolic acidosis Cardiac arrest	Susceptible: CF, FOX, TET, TMP-SMX, TOB Intermediate: AMK Resistant: AMP, PIP	Died 24 h after admission
[14]	Germany	Newborn	Male	Maternal history: Severe respiratory infection a few weeks before delivery Exposure: Both parents frequently consumed smoked coalfish	Fever (38.3 °C) Jaundice Severe dyspnea Protruding abdomen Prolonged capillary refill Muscular hypertension Hyperirritability Extensor tonus Thrombocytopenia Multilocular lysis Hydrocephalus ex vacuo Brain necrosis with bleeding Convulsive attacks Myoclonus with oral automatisms Respiratory failure	Susceptible: MEZ, PIP, CLX, CXM, CTX, NET, GEN, OFX, CIP, IPM, TMP-SMX Resistant: AMP	Respiratory support withdrawn at 51 days; infant death implied
[15]	Belgium	65	Male	Exposure: Ingested raw mussels	Chills and sweating Fever (39.5 °C) Tachypnea Hepatomegaly Uniformly tan skin Right lower limb: cellulitis with bullae and purpura Leukopenia High serum ferritin Confusion Hip arthritis and proximal tibial osteitis Primary hemochromatosis	Resistant: AMP Susceptible: all others tested	Not mentioned
[10]	Hong Kong	13	Female	Acute promyelocytic leukemia in first complete remission Allogeneic bone marrow transplant (matched siblings) Conditioning: Busulfan + cyclophosphamide Prophylaxis: TMP-SMX pre-infusion; acyclovir + fluconazole only during transplant	Fever Chills Vomiting	Susceptible: CXM, CAZ, GEN, TET, CIP Resistant: AMP	Recovered
[16]	Malaysia	6	Female	Induction chemotherapy for acute lymphoblastic leukemia Achieved remission	Fever Malaise, lethargy Watery diarrhea Abdominal pain Hypotensive Anemia Thrombocytopenia	Susceptible: TMP-SMX, CAZ, CXM, AMP-SUL, NET, GEN, CIP Resistant: AMP	Recovered
[17]	Sweden	67	Male	Multiple myeloma: treated with melphalan and prednisone Repeated blood transfusions for anemia Exposure: Pricked left thumb while fishing (freshwater lake)	Swollen and reddened left thumb Lymphangitis Painful axillary lymph node Elevated CRP Anemia Leukopenia Thrombocytopenia	Susceptible: CXM, CHL, CIP, TMP-SMX, GEN, IPM	Recovered
[18]	Canada	48 h old	Male	Birth: Labor induced at 36 weeks Right leukocoria Maternal history: Severe diarrhea for 2 weeks before delivery after eating raw oysters	Fever Irritability with poor feeding Respiratory distress Xanthochromia Leukocytosis Elevated protein Endophthalmitis (right eye) Bilateral enlarging frontal and occipital abscesses Communicating hydrocephalus	Not mentioned	Recovered Long-term sequelae
[19]	USA	13	Female	Sickle cell disease (diagnosed at birth) 3 prior hospitalizations and 3 blood transfusions Prophylaxis and vaccines: Penicillin; Pneumovax 8 months prior Exposure: Ate crab legs at a crab buffet 4 days before admission	Fever (39.4 °C) Sore throat Abdominal and leg pain Multiple nonbilious, non-bloody vomiting Hypotension Metabolic acidosis Anemia Leukocytosis Elevated transaminases Extensive bilateral infiltrates Ventricular fibrillation Septic shock DIC Renal insufficiency Hepatic dysfunction Pneumothorax Splenic infarct	Not mentioned	Recovered
[20]	Greece	34	Female	Thalassemia intermedia Splenectomy at 13 years Maintained stable blood counts; no regular transfusions Medications: Hydroxyurea 1 g/day from age 29 Oral deferiprone (L1) started at age 32; ferritin taken over 21 months, then stopped	Fever (40.2 °C) Almost unconscious Abdominal cramping Nausea Vomiting Watery, non-mucoid, non-bloody, greenish-yellow, foamy diarrhea Chills, sweating Sepsis, shock Pallor and slight jaundice Disorientation DIC Anemia Leukocytosis with lymphocytosis Thrombocytopenia Decreased serum antibodies Low total serum protein Low complement levels (C3, C4)	Susceptible: AMP, TIC, PIP, ATM, MEM, CAZ, GEN, TOB, AMK, NET, TET, CHL, TMP-SMX, PEF, CIP	Died 24 h after ICU admission
[21]	Brazil	16	Female	β^0^-thalassemia carrier Medications: Benzathine penicillin every 21 days; folic acid supplementation	Fever Lumbar and lower limb pain Vomiting Multiple diarrhea (non-bloody, non-mucoid, non-purulent) Pallor and dehydration Slight jaundice Hypotension and tachycardia Urinary bacteriuria with pyuria Anemia Leukocytosis Elevated liver enzymes and ferritin Metabolic acidosis Hepatomegaly Bilateral pleural effusions	Susceptible: AMK, GEN, TMP-SMX, IPM, CIP, CTX Resistant: AMP	Recovered
[22]	USA	51	Male	HBV-associated liver cirrhosis Treatment: Lamivudine + tenofovir Diabetes mellitus (metformin) Donor history: Teenage boy drowned in freshwater lake	No intraoperative complications occurred *P. shigelloides* isolated from donor blood cultures before organ procurement (informed day 1 post-LT)	Susceptible: CIP Resistant: third- and fourth-generation cephalosporins	Recovered
[23]	USA	71	Male	Exposure: 2 weeks in Swiss Alps—hunting and consuming game Anemia of unclear etiology (treated with erythropoietin) Diabetes (pioglitazone + glyburide) Coronary artery stents	Chest pain and shortness of breath Severe headaches Abdominal discomfort Extreme fatigue Hypothermia (35.5 °C) Elevated BP Tachycardia Generalized petechial rash Cyanosis of face and extremities Oxygen saturation 60% Bilateral infiltrates, pulmonary edema Anemia, renal and liver dysfunction, hyperkalemia, elevated troponin and LDH, lactic acidosis	Not mentioned	Died 3 h after presentation
[24]	USA	43	Male	Homozygous hereditary hemochromatosis; pyruvate kinase deficiency Splenectomy (age 4) Exposures: New puppy, home-cooked clams, frequent swimming (Lake Erie)	Fever Chills Weakness Septic shock Multiorgan failure DIC Lactic acidosis Macrocytic anemia Hemodynamic collapse	Susceptible: FEP, CTX, CIP, GEN, IPM, LEV, PIP-TZB, TOB, TMP-SMX	Died on hospital day 3
[25]	USA	55	Male	Mechanical aortic and mitral valve replacements Prior endocarditis Exposure: Fishing in the Mississippi River	Rapid respiratory failure Refractory septic shock Multiorgan failure Severe hypotension Whole-body blue discoloration from methylene blue infusion	Susceptible: empiric antibiotics	Died following withdrawal of care due to progressive multiorgan failure
[26]	Canada	24	Female	Pregnancy: 11 weeks gestation Previous chlamydial cervicitis (treated) Active smoker	Septic shock Needed dilatation and curettage Elevated CRP Elevated liver enzymes Chorioamnionitis, necrotizing deciduitis, villitis	Susceptible: CTX, CAZ, AMC, PIP-TZB, CIP, TMP-SMX, TOB, ETP Intermediate: CFZ, GEN Resistant: AMP	Recovered
[27]	USA	78	Female	Hypertension Hyperlipidemia Multivessel CAD CABG Stents Sick sinus syndrome Atrial flutter Pacemaker Repaired rectovaginal fistula	Severe leukocytosis Lactic acidosis Acute colitis Mesenteric ischemia Necrosis of small bowel and entire colon Splenectomy Subtotal colectomy Ileostomy Secondary VRE UTI	Susceptible: FEP, LEV, CIP, MEM, CTX, PIP-TZB	Recovered
[28]	USA	80	Female	Hypertension Asthma Hypothyroidism Exposure: boating in fresh river water	Cellulitis to severe sepsis Acute hypoxic respiratory failure Altered mentation Hypotension Surgical debridement	Susceptible: CTX, CIP, ETP, MEM	Recovered
[29]	Japan	49	Male	Alcoholic cirrhosis (Child–Pugh B) Chronic alcohol and long-term smoking Exposure: Ingested dojo nabe	Septic shock Multiorgan dysfunction Acute kidney injury Respiratory failure Infective enterocolitis	Susceptible: AMP-SUL, PIP-TZB, CTX, CAZ, FEP, ATM, MEM, AMK, LEV Resistant: AMP	Recovered
[30]	Japan	77	Male	Stage IIIC hilar cholangiocarcinoma with postoperative recurrence Left and caudate lobectomy, bile duct resection, biliary reconstruction Ongoing chemotherapy (gemcitabine + fluorouracil) Hypertension	Mixed bacteremia Intratumoral abscess Recurrent fever Gastrointestinal bleeding Subsequent candidemia (*Candida albicans*)	Susceptible: AMP-SUL, PIP-TZB, CFZ, CMZ, CTX, CAZ, FEP, IPM, MEM, ATM, CIP, LEV Resistant: AMP, PIP	Survived acute infection
[31]	Australia	74	Male	Well-controlled epilepsy Hypertension Hyperlipidemia Asthma, COPD Steatohepatitis Exposure: Ingested fermented pork sausage and raw seafood	Infective endocarditis Severe mitral regurgitation Heart failure features Recurrent fever Cholecystitis	Susceptible: AMC, CTX, CIP	Recovered

CRP = C-reactive protein; COPD = chronic obstructive pulmonary disease; LT = liver transplant; LDH = lactate dehydrogenase; BP = blood pressure; CAD = coronary artery disease; CABG = coronary artery bypass graft; DIC = disseminated intravascular coagulation; VRE UTI = vancomycin-resistant Enterococcus urinary tract infection; AMP = ampicillin; TIC = ticarcillin; PIP = piperacillin; PIP-TZB = piperacillin–tazobactam; AMC = amoxicillin–clavulanate; AMP-SUL = ampicillin–sulbactam; CFZ = cefazolin; CMZ = cefmetazole; FOX = cefoxitin; CXM = cefuroxime; CAZ = ceftazidime; CTX = cefotaxime; FEP = cefepime; ATM = aztreonam; MEM = meropenem; IPM = imipenem; ETP = ertapenem; GEN = gentamicin; TOB = tobramycin; AMK = amikacin; NET = netilmicin; TET = tetracycline; CHL = chloramphenicol; TMP-SDZ = trimethoprim–sulfadiazine; TMP-SMX = trimethoprim–sulfamethoxazole; PEF = pefloxacin; CIP = ciprofloxacin; LEV = levofloxacin.

**Table 2:** Patient characteristics, predisposing factors, and clinical presentation.

Characteristics	All Patients (*n* = 22)	Survived (*n* = 16)	Died (*n* = 6)
Age, Median (IQR)	46 (15.5–68)	50 (14–70.5)	38.5 (32–55)
Male gender, *n* (%)	13 (59.1)	8 (50)	5 (83.3)
Predisposing factors			
Seafood ingestion (raw or undercooked), *n* (%)	8 (36.3)	6 (37.5)	2 (33.3)
Freshwater exposure, *n* (%)	7 (27.3)	3 (18.8)	3 (50)
Hypertension, *n* (%)	4 (18.2)	4 (25)	0
Active chemotherapy or cytotoxic therapy, *n* (%)	4 (18.2)	4 (25)	0
Asplenia, *n* (%)	3 (13.6)	0	3 (50)
Cardiovascular disease, *n* (%)	3 (13.6)	2 (12.5)	1 (16.7)
Hematological malignancy (leukemia, myeloma), *n* (%)	3 (13.6)	3 (18.8)	0
Chronic liver disease including cirrhosis, *n* (%)	3 (13.6)	3 (18.8)	0
Iron overload, *n* (%)	3 (13.6)	1 (6.3)	2 (33.3)
Pregnancy-associated infection, *n* (%)	3 (13.6)	2 (12.5)	1 (16.7)
Hemoglobinopathy—thalassemia syndromes, *n* (%)	2 (9.1)	1 (6.3)	1 (16.7)
Hemoglobinopathy—sickle cell disease, *n* (%)	2 (9.1)	2 (12.5)	0
Diabetes, *n* (%)	2 (9.1)	1 (6.3)	1 (16.7)
Prosthetic valve, *n* (%)	1 (4.5)	0	1 (16.7)
Clinical Presentation			
Fever, *n* (%)	14 (63.6)	10 (62.5)	4 (66.7)
Septic shock, sepsis, *n* (%)	8 (36.3)	4 (25)	4 (66.7)
Vomiting, *n* (%)	7 (31.8)	5 (31.3)	2 (33.3)
Anemia, *n* (%)	7 (31.8)	4 (25)	3 (50)
Diarrhea, *n* (%)	6 (27.3)	5 (31.3)	1 (16.7)
Thrombocytopenia, *n* (%)	6 (27.3)	3 (18.8)	3 (50)
Chills, *n* (%)	5 (22.7)	3 (18.8)	2 (33.3)
Multiorgan failure, *n* (%)	5 (22.7)	2 (12.5)	3 (50)
Neurologic involvement (seizure, encephalitis, and AMS), *n* (%)	3 (13.6)	2 (12.5)	1 (16.7)
DIC, *n* (%)	3 (13.6)	1 (6.3)	2 (33.3)
Soft tissue infection, *n* (%)	3 (13.6)	3 (18.8)	0
Hypotension, *n* (%)	2 (9.1)	2 (12.5)	0
Enterocolitis, *n* (%)	1 (4.5)	1 (6.3)	0

## Data Availability

The original contributions presented in this study are included in the article/Supplementary Materials. Further inquiries can be directed to the corresponding author.

## Supplementary Materials

The following supporting information can be downloaded at: https://www.mdpi.com/article/10.3390/pathogens15010123/s1. Supplementary Table S1. Preferred Reporting Items for Systematic Reviews and Meta-Analyses extension for Scoping Reviews (PRISMA-ScR) Checklist; Supplementary Table S2. Searching Strategy for *Plesiomonas* Bloodstream Infections.

## Abbreviations

The following abbreviations are used in this manuscript:

IQR	interquartile range
CAD	coronary artery disease
DIC	disseminated intravascular coagulation
AMS	altered mental status
MALDI-TOF	matrix-assisted laser desorption/ionization time-of-flight

## References

[1] Michael Janda J., Abbott S.L., McIver C.J. (2016). *Plesiomonas shigelloides* Revisited. Clin. Microbiol. Rev..

[2] Cortés-Sánchez A.D.J., Espinosa-Chaurand L.D., Díaz-Ramirez M., Torres-Ochoa E. (2021). *Plesiomonas*: A Review on Food Safety, Fish-Borne Diseases, and Tilapia. Sci. World J..

[3] Chen X., Chen Y., Yang Q., Kong H., Yu F., Han D., Zheng S., Cui D., Li L. (2013). *Plesiomonas shigelloides* Infection in Southeast China. PLoS ONE.

[4] Skwor T. (2025). *Aeromonas* and *Plesiomonas*. Microorganisms.

[5] Ellner P.D., McCarthy L.R. (1973). *Aeromonas shigelloides* Bacteremia: A Case Report. Am. J. Clin. Pathol..

[6] Woo P.C.Y., Lau S.K.P., Yuen K.Y. (2005). Biliary Tract Disease as a Risk Factor for *Plesiomonas shigelloides* Bacteraemia: A Nine-Year Experience in a Hong Kong Hospital and Review of the Literature. New Microbiol..

[7] Khan F.A., Fisher M.A., Khakoo R.A. (2007). Association of Hemochromatosis with Infectious Diseases: Expanding Spectrum. Int. J. Infect. Dis..

[8] Henderson D.P., Wyckoff E.E., Rashidi C.E., Verlei H., Oldham A.L. (2001). Characterization of the *Plesiomonas shigelloides* Genes Encoding the Heme Iron Utilization System. J. Bacteriol..

[9] Yeh T.J., Tsai W.C. (1991). *Plesiomonas shigelloides*-Associated Diarrhea. Chin. Med. J..

[10] Lee A.C.W., Yuen K.Y., Ha S.Y., Chiu D.C.K., Lau Y.L. (1996). *Plesiomonas shigelloides* Septicemia: Case Report and Literature Review. Pediatr. Hematol. Oncol..

[11] Ingram C.W., Morrison A.J., Levitz R.E. (1987). Gastroenteritis, Sepsis, and Osteomyelitis Caused by *Plesiomonas shigelloides* in an Immunocompetent Host: Case Report and Review of the Literature. J. Clin. Microbiol..

[12] Paul R., Siitonen A., Kärkkäinen P. (1990). *Plesiomonas shigelloides* Bacteremia in a Healthy Girl with Mild Gastroenteritis. J. Clin. Microbiol..

[13] Clark R.B., Westby G.R., Spector H., Soricelli R.R., Young C.L. (1991). Fatal *Plesiomonas*-*shigelloides* septicemia in a splenectomized patient. J. Infect..

[14] Terpeluk C., Goldmann A., Bartmann P., Pohlandt F. (1992). *Plesiomonas shigelloides* Sepsis and Meningoencephalitis in a Neonate. Eur. J. Pediatr..

[15] Delforge M.L., Devriendt J., Glupczynski Y., Hansen W., Douat N. (1995). *Plesiomonas shigelloides* Septicemia in a Patient with Primary Hemochromatosis. Clin. Infect. Dis..

[16] Riley P.A., Parasakthi N., Abdullah W.A. (1996). *Plesiomonas shigelloides* Bacteremia in a Child with Leukemia. Clin. Infect. Dis..

[17] Jönsson I., Monsen T., Wiström J. (1997). A Case of *Plesiomonas shigelloides* Cellulitis and Bacteraemia from Northern Europe. Scand. J. Infect. Dis..

[18] Marshman W.E., Lyons C.J. (1998). Congenital Endophthalmitis Following Maternal Shellfish Ingestion. Aust. N. Z. J. Ophthalmol..

[19] Ampofo K., Graham P., Ratner A., Rajagopalan L., Della-Latta P., Saiman L. (2001). *Plesiomonas shigelloides* Sepsis and Splenic Abscess in an Adolescent with Sickle-Cell Disease. Pediatr. Infect. Dis. J..

[20] Tzanetea R., Konstantopoulos K., Xanthaki A., Kalotychou V., Spiliopoulou C., Michalopoulos A., Rombos Y. (2002). *Plesiomonas shigelloides* Sepsis in a Thalassemia Intermedia Patient. Scand. J. Infect. Dis..

[21] Auxiliadora-Martins M., Bellissimo-Rodrigues F., Viana J.M., Teixeira G.C.A., Nicolini E.A., Cordeiro K.S.M., Colozza G., Martinez R., Martins O.A., Basile A. (2010). Septic Shock Caused by *Plesiomonas shigelloides* in a Patient with Sickle Beta-Zero Thalassemia. Heart Lung.

[22] Bonatti H., Sifri C., Sawyer R.G. (2012). Successful Liver Transplantation from Donor with *Plesiomonas shigelloides* Sepsis after Freshwater Drowning: Case Report and Review of Literature on Gram-Negative Bacterial Aspiration during Drowning and Utilization of Organs from Bacteremic Donors. Surg. Infect..

[23] Okon E., Bishburg E., Ugras S., Chan T., Wang H. (2013). *Clostridium perfringens* Meningitis, *Plesiomonas shigelloides* Sepsis: A Lethal Combination. Am. J. Case Rep..

[24] Samannodi M., Zhao A., Nemshah Y., Shiley K. (2016). *Plesiomonas shigelloides* Septic Shock Leading to Death of Postsplenectomy Patient with Pyruvate Kinase Deficiency and Hemochromatosis. Case Rep. Infect. Dis..

[25] Varghese C., Demartino E., Ashley E., Jacobi J., Lisa D., Sharain K., Clain J. (2016). Methylene Blue in Refractory Septic Shock: Losing Our Way for the Sake of a Map?. Crit. Care Med..

[26] Cornut G., Marchand-Senecal X., Gaudreau C., Berdugo J., Gariepy G., Tremblay C., Savard P. (2017). *Plesiomonas shigelloides*: An Unusual Cause of Septic Abortion. Case Rep. Infect. Dis..

[27] McDonald A.E. (2020). A Case of *Plesiomonas* Bacteremia Without Reported Freshwater Exposure. HCA Healthc. J. Med..

[28] Pennycook K.M., Pennycook K.B., McCready T.A., Kazanowski D. (2020). Severe Cellulitis and Bacteremia Caused by *Plesiomonas shigelloides* Following a Traumatic Freshwater Injury. IDCases.

[29] Shinohara T., Okamoto K., Koyano S., Otani A., Yamashita M., Wakimoto Y., Jubishi D., Hashimoto H., Ikeda M., Harada S. (2021). *Plesiomonas shigelloides* Septic Shock Following Ingestion of Dojo Nabe (Loach Hotpot). Open Forum Infect. Dis..

[30] Itoh N., Akazawa N., Yanaidani T., Kuwahara T. (2022). Clinical and Microbiological Features of Intratumor Abscess with Bloodstream Infection Caused by *Plesiomonas shigelloides*, *Citrobacter freundii*, *Streptococcus mitis*/*oralis*, *Clostridium perfringens*, and *Candida albicans* in a Patient with Cholangiocarcinoma: A Case Report. J. Infect. Chemother..

[31] Rao A.C.A., Senanayake S., Khan L. (2025). “Something Fishy Going On…”; First Case Report of *Plesiomonas shigelloides* Endocarditis Manifesting as a Seizure. Eur. Heart J. Case Rep..

[32] Herrera F.C., Santos J.A., Otero A., García-López M.L. (2006). Occurrence of *Plesiomonas shigelloides* in Displayed Portions of Saltwater Fish Determined by a PCR Assay Based on the HugA Gene. Int. J. Food Microbiol..

[33] Abuga K.M., Muriuki J.M., Williams T.N., Atkinson S.H. (2020). How Severe Anaemia Might Influence the Risk of Invasive Bacterial Infections in African Children. Int. J. Mol. Sci..

[34] Ekundayo T.C., Okoh A.I. (2018). Pathogenomics of Virulence Traits of *Plesiomonas shigelloides* That Were Deemed Inconclusive by Traditional Experimental Approaches. Front. Microbiol..

[35] Hasa E., Hartmann P., Schnabl B. (2022). Liver Cirrhosis and Immune Dysfunction. Int. Immunol..

[36] Brandhagen D.J., Alvarez W., Therneau T.M., Kruckeberg K.E., Thibodeau S.N., Ludwig J., Porayko M.K. (2000). Iron Overload in Cirrhosis—HFE Genotypes and Outcome after Liver Transplantation. Hepatology.

[37] Tsafaras G.P., Ntontsi P., Xanthou G. (2020). Advantages and Limitations of the Neonatal Immune System. Front. Pediatr..

[38] Cavaliere A.F., Perelli F., Mattei A., Dal Poggetto P., Marchi L., Vidiri A., Turrini I., Aquilini D., Brunelli T., Scambia G. (2023). Case Report: Vertical Transmission of *Plesiomonas shigelloides*. Is It Time to Strengthen Information on Safety Concerns for Raw Seafood Dietary Exposure in Pregnancy?. J. Matern.-Fetal Neonatal Med..

[39] Cleary T.G. (2008). *Plesiomonas* *shigelloides*. Principles and Practice of Pediatric Infectious Disease.

[40] Chuang T., Desai K., Adhyaru B. (2020). Living in the Creeks. J. Gen. Intern. Med..

[41] Magana-Arachchi D.N., Wanigatunge R.P. (2020). Ubiquitous Waterborne Pathogens. Waterborne Pathogens: Detection and Treatment.

[42] Stock I., Wiedemann B. (2001). Natural Antimicrobial Susceptibilities of *Plesiomonas shigelloides* Strains. J. Antimicrob. Chemother..

[43] Oeschger T., Kret L., Erickson D. (2022). Multiplexed Paper-Based Assay for Personalized Antimicrobial Susceptibility Profiling of Carbapenem-Resistant *Enterobacterales* Performed in a Rechargeable Coffee Mug. Sci. Rep..

[44] Smit C.C.H., Keighley C., Rogers K., Miyakis S., Taxis K., Sanderson-Smith M., Nicholas N., Robertson H., Pont L.G. (2025). Geo-Temporal Variation in the Antimicrobial Resistance of *Escherichia coli* in the Community. Antibiotics.

[45] Iskandar K., Molinier L., Hallit S., Sartelli M., Hardcastle T.C., Haque M., Lugova H., Dhingra S., Sharma P., Islam S. (2021). Surveillance of Antimicrobial Resistance in Low- and Middle-Income Countries: A Scattered Picture. Antimicrob. Resist. Infect. Control.

[46] Woods W.A., Watson M., Ranaweera S., Tajuria G., Sumathipala A. (2023). Under-Representation of Low and Middle Income Countries (LMIC) in the Research Literature: Ethical Issues Arising from a Survey of Five Leading Medical Journals: Have the Trends Changed?. Glob. Public Health.

[47] Elbehiry A., Aldubaib M., Abalkhail A., Marzouk E., Albeloushi A., Moussa I., Ibrahem M., Albazie H., Alqarni A., Anagreyyah S. (2022). How MALDI-TOF Mass Spectrometry Technology Contributes to Microbial Infection Control in Healthcare Settings. Vaccines.

