# Supplementary material for: Plesiomonas shigelloides Bacteremia: A Scoping Review of Epidemiology, Clinical Characteristics, Outcomes, and Implications of Antimicrobial Stewardship
Source: Pathogens. 2026 Jan 22;15(1):123. doi: 10.3390/pathogens15010123 (PMC12845026; doi:10.3390/pathogens15010123)
Supplement: Supplementary file 1 [file pathogens-15-00123-s001.zip › PRISMA-ScR for Plesiomonas shigelloides bacteremia - updated.pdf]

Supplemetary Table S1. Preferred Reporting Items for Systematic Reviews and Meta-Analyses extension for Scoping Reviews (PRISMA-ScR) Checklist

SECTION	ITEM	PRISMA-ScR CHECKLIST ITEM	REPORTED ON PAGE #
TITLE	1	*Plesiomonas shigelloides* Bacteremia: A Scoping Review of Epidemiology, Clinical Characteristics, Outcomes, and Implications of Antimicrobial Stewardship	Page 1
ABSTRACT			
Structured summary	2	Abstract: *Plesiomonas shigelloides* bacteremia	Page 1
INTRODUCTION			
Rationale	3	Existing reviews often aggregate enteric and extraintestinal infections or are limited by dated literature, incomplete case capture, or a lack of systematic methodology. Given the continued emergence of atypical presentations, potential emerging antimicrobial resistance, and diagnostic methods, a more recent and structured overview of *P. shigelloides* bacteremia is warranted.	Page 2
Objectives	4	This scoping review aimed to collate peer-reviewed research published in the literature to clarify the epidemiology, host risk factors, clinical manifestations, antimicrobial susceptibility patterns, and outcomes of *P. shigelloides* bacteremia. This study identifies knowledge gaps and patterns that are not apparent in earlier case reports, providing direction for clinical awareness. By	Page 2

		synthesizing evidence across decades of research, this review aimed to enhance understanding of this rare but important infection.	
METHODS			
Protocol and registration	5	The protocol was registered on Open Science Framework (Registration DOI: 10.17605/OSF.IO/M64X9).	Page 2
Eligibility criteria	6	Inclusion criteria for this review are as follows: (1) *Plesiomonas* bloodstream infection; (2) human patient; (3) case report, case series, or cohort studies; (4) full-text available; and (5) written in English. No year filter was used for this review.	Page 3
Information sources	7	PubMed, Scopus, Web of Science, and EMBASE	Page 2
Search	8	The keyword "*Plesiomonas*" was combined with outcome-related terms using AND, while related terms ("bacteremia" OR "bloodstream infection") were combined using OR, with parentheses applied to maintain a consistent query structure across databases. Full search strategies are provided in Supplementary Material 2. The searches were conducted on 8th December 2025.	Supplementary Materials
Selection of sources of evidence	9	Titles and abstracts were screened independently by two authors to identify potentially relevant studies, followed by a full-text review to confirm eligibility.	Page 3
Data charting process	10	Data extraction was independently performed	Page 3

		by the authors using a standardized Excel spreadsheet, with discrepancies resolved by consensus to ensure consistency and minimize bias.	
Data items	11	Extracted data included epidemiology, host risk factors, clinical manifestations, antimicrobial susceptibility patterns, and outcomes of *P. shigelloides* bacteremia.	Page 3
Critical appraisal of individual sources of evidence	12	Critical appraisal was not done as this is a scoping review. A narrative synthesis was conducted for this scoping review.	Page 3
Synthesis of results	13	A narrative synthesis was conducted for this scoping review.	Page 3
RESULTS			
Selection of sources of evidence	14	A total of 172 articles were screened; 22 met eligibility criteria and were included. Exclusions were due to duplication or did not meet criteria	Page 3
Characteristics of sources of evidence	15	Extracted data included epidemiology, host risk factors, clinical manifestations, antimicrobial susceptibility patterns and outcomes of *P. shigelloides* bacteremia. Full citations are included in reference list.	Page 5 to 14
Critical appraisal within sources of evidence	16	Critical appraisal was not done as this is a scoping review	
Results of individual sources of evidence	17	Data on the epidemiology, host risk factors, clinical manifestations, antimicrobial susceptibility patterns and outcomes of *P. shigelloides* bacteremia	Page 5 to 14

		were tabulated in separate tables and figures to address the objectives.	
Synthesis of results	18	Tabulated data summarized epidemiology, host risk factors, clinical manifestations, antimicrobial susceptibility patterns and outcomes of *P. shigelloides* bacteremia to address the objectives	Table 1 and 2
DISCUSSION			
Summary of evidence	19	Summary of main results and linking key results with known relevant evidence.	Page 14 to 16
Limitations	20	As a scoping review of published case reports, this study is methodologically limited by an inability to establish causality or provide definitive population-level incidence and outcome data.	Page 16
Conclusions	21	These findings reinforce the importance of early recognition, antimicrobial stewardship, and cautious interpretation of outcomes based on rare, case-based evidence.	Page 17
Funding			
Funding	22	No external funding was received for this review	Page 17
