# Supplementary material for: Plesiomonas shigelloides Bacteremia: A Scoping Review of Epidemiology, Clinical Characteristics, Outcomes, and Implications of Antimicrobial Stewardship
Source: Pathogens. 2026 Jan 22;15(1):123. doi: 10.3390/pathogens15010123 (PMC12845026; doi:10.3390/pathogens15010123)
Supplement: Supplementary file 1 [file pathogens-15-00123-s001.zip › Table S2 Searching Strategy for Plesiomonas Bloodstream Infections - updated.pdf]

Table S2 Searching Strategy for Plesiomonas Bloodstream Infections

**PubMed**
("Plesiomonas"[Mesh] OR Plesiomonas[tiab]) AND ("Bacteremia"[Mesh] OR bacteremia[tiab] OR "Bloodstream Infection"[tiab] OR "Bloodstream Infections"[tiab])
**Scopus**
(TITLE-ABS-KEY (Plesiomonas) OR TITLE-ABS (Plesiomonas)) AND (TITLE-ABS-KEY (bacteremia) OR TITLE-ABS-KEY (bloodstream infection) OR TITLE-ABS-KEY (bloodstream infections))
**Web of Science**
TS=(Plesiomonas)<br>AND<br>TS=(bacteremia OR "bloodstream infection" OR "bloodstream infections")
**Embase**
('plesiomonas'/exp OR 'plesiomonas':ti,ab) AND ('bacteremia'/exp OR bacteremia:ti,ab OR 'bloodstream infection':ti,ab OR 'bloodstream infections':ti,ab)
